# Effect of Management Strategies on Ecosystem C:N:P Stoichiometry and Stocks in the Semi-Arid Grasslands of Northern China

**DOI:** 10.3390/plants15101480

**Published:** 2026-05-12

**Authors:** Xiaoguang Xing, Huailiang Wang, Bin Liu, Fengchan Liu, Jingyi Xu, Huitao Shen

**Affiliations:** 1 Water Resources Research and Water Conservancy Technology Test and Extension Center of Hebei, Shijiazhuang 050061, China; 2 Hebei Technology Innovation Center for Geographic Information Application, Institute of Geographical Sciences, Hebei Academy of Sciences, Shijiazhuang 050021, China; 3 Station of Agro-Pastoral Ecotone in Bashang Hebei (Grassland), Ecological Quality Comprehensive Monitoring Station, Ministry of Ecology and Environment of People’s Republic of China, Chengde 068462, China

**Keywords:** nutrients, ecological stoichiometry, stocks, semi-arid grassland, management strategies

## Abstract

Grassland management strategies profoundly influence ecosystem nutrient dynamics, yet how long-term practices affect carbon (C), nitrogen (N), and phosphorus (P) stoichiometry and stocks in plant–soil systems remains poorly understood, particularly in semi-arid regions. This study evaluated the effects of different management strategies—enclosing (EG), grazing (GG), and mowing with enclosure (MG)—on C, N, and P concentrations, stoichiometric ratios, and stocks in both plant and soil across a semi-arid grassland of northern China. Our results showed that GG led to higher N and P concentrations in plant tissues, while EG enhanced C concentrations and resulted in the highest ecosystem C and N stocks. The N:P ratios under EG and GG (15.3 and 15.6) suggested co-limitation by N and P, while the lower ratio under MG (11.8) indicated stronger N limitation. The concentrations of soil C, N, and P were greatest under EG and declined with depth. Soil C:N ratios remained stable across treatments and below 12. The N:P ratios ranged from 1.78 to 3.81 across all treatments and soil depths, and were significantly elevated under EG. Strong positive correlations were observed among soil C, N, and P, and between soil and plant nutrient pools. Total P stocks were unaffected by management, reflecting the geogenic origin of P. These findings highlight that enclosing is the most effective strategy for enhancing C and N stocks in semi-arid grasslands, while also revealing tight plant–soil nutrient coupling and the relative stability of P dynamics under different management regimes.

## 1. Introduction

Grassland ecosystems cover around 52.5 million km^2^, representing roughly 40.5% of the Earth’s land surface, excluding Antarctica and Greenland [1]. They provide numerous critical services to human societies, such as forage production, livestock biomass, water and nutrient regulation, and soil CO_2_ sequestration [2,3]. Grassland management—essentially the transformation of natural ecosystems into managed units—is a primary means of meeting human demands [4,5]. These management strategies can substantially alter ecosystem function and structure, with notable consequences for plant productivity and nutrient dynamics [6,7].

Carbon (C), nitrogen (N), and phosphorus (P) are three major nutrients for plant growth, and these three elements are tightly interrelated in their biological and geochemical processes [8,9]. Critical knowledge gaps persist regarding how long-term grassland management affects plant tissue elements, and whether shifts in plant nutrients stoichiometries (e.g., C:N:P ratios) are linked to corresponding changes in soil C, N, and P stocks and their stoichiometric ratios [10]. Therefore, understanding the response of C:N:P stoichiometry to management strategies is important for evaluating human-induced effects on ecosystem functions and formulating sustainable approaches to managing grassland ecosystems [2,3].

Grazing, mowing and enclosing are the predominant grassland management practices worldwide [5,7], and their impacts on C, N, and P dynamics are central to understanding these human-induced changes [11]. Numerous studies have demonstrated that grazing indirectly influences the cycling of soil C, N, and P in grassland ecosystems by altering biomass production and allocation, as well as through the effects of livestock urine and feces deposition [12,13,14]. Compared with grazing, mowing is characterized by non-selective biomass harvesting and the absence of trampling and excreta input, resulting in unique effects on soil nutrient dynamics [15]. Moderate mowing could enhance soil C and N concentrations and subsequently alter nutrient cycling [16]. However, Hu et al. [11] reported that uniform biomass removal through mowing may reduce grassland C sequestration through changes in plant community structure and the nutrient cycling processes driven by plants. Enclosure establishment has been increasingly adopted worldwide as a strategy for restoring degraded grasslands [11,17]. According to Abrigo et al. [18] and Dai et al. [19], enclosure may enhance soil C and N stocks. Nevertheless, such effects are not necessarily sustained over time [20], and long-term protection may lead to negative outcomes [21,22]. These inconsistent and even contradictory findings suggest that the impacts of different management strategies may vary depending on site-specific conditions and temporal scales.

Given this context-dependency, it is essential to examine these effects in specific, representative regions [2,12]. Grasslands cover approximately 40% of China’s total national land, representing about 6–8% of the world’s grassland area and holding considerable ecological significance at both regional and global scales [23]. Within this region, the agro-pastoral transitional zone of northern China (APTZNC) serves as a critical ecotone between grazing and farming systems [24,25]. Pan et al. [26] explored how different management regimes affect soil properties, including microbial biomass C, N, P and their stoichiometric ratios. Nevertheless, studies comparing C, N, and P stoichiometry in the plant–soil system across management strategies in the APTZNC are still scarce. To address this gap, we conducted a field experiment examining C, N, and P stocks and their stoichiometric ratios in both aboveground and belowground pools under three management strategies in this region. The three strategies were enclosure control grassland (EG, with no grazing or mowing activities since early 2019), grazing grassland (GG, free grazing by domestic livestock throughout the year), and mowing combined with enclosure grassland (MG, mowing in September every year with biomass removed since 2019), respectively. Our findings on C, N, and P stoichiometry under these management strategies have important implications for greenhouse gas (GHG) mitigation in grassland systems, as they help clarify how management practices influence carbon and nutrient cycling and thus the potential for soil carbon sequestration and emission reduction. Specifically, we focused on two main questions: (1) How are plant C:N:P ratios impacted by different grassland management strategies? (2) Which management strategy most favors the accumulation of ecosystem C, N, and P?

## 2. Results

### 2.1. Soil Nutrients Concentrations and Stoichiometries

Among the three management strategies, the concentrations of soil C, N, and P were highest under EG and lowest under MG. Concentrations of all three elements decreased as the soil layer deepened (Table 1). Across all soil layers, soil C and N concentrations differed significantly among the three management strategies (*p* < 0.05). For soil P, the three strategies differed significantly in the 0–20 cm soil layer (*p* < 0.05), except for the 20–40 cm depth.

The soil C:N ratio remained below 12 across all three management strategies. The C:N ratio, in contrast to soil C and N concentrations, exhibited no significant variation within each grassland management treatment over the 0–40 cm soil layers. Among the three strategies, MG exhibited the lowest soil C:P ratio. Across soil layers, the C:P ratio decreased with increasing soil depth. Additionally, EG exhibited a significantly higher soil N:P ratio compared with the other two management regimes (*p* < 0.05).

### 2.2. Nutrients Concentrations and Stoichiometric Ratios in Plant Layers

**Figure 1:**
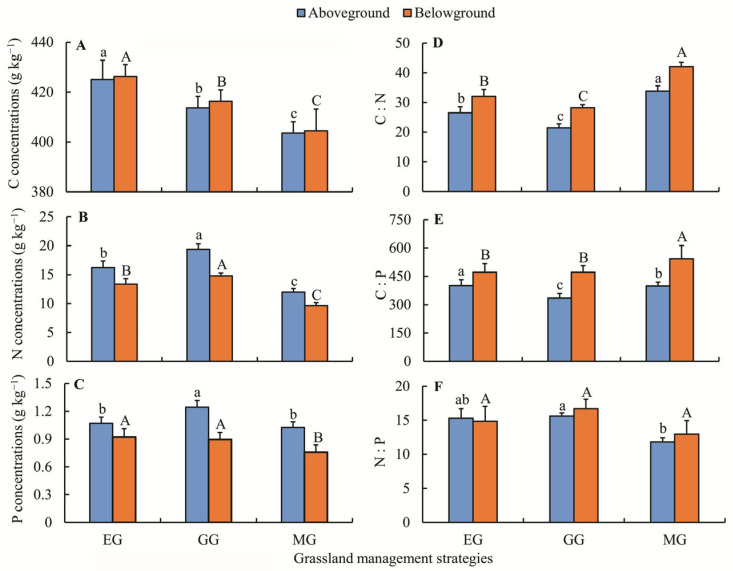
Concentrations of C, N, P, and their stoichiometric ratios under different management strategies. (**A**) C concentrations; (**B**) N concentrations; (**C**) P concentrations; (**D**) C:N; (**E**) C:P; (**F**) N:P. Different lowercase letters denote significant differences (*p* < 0.05) among strategies for aboveground components, while uppercase letters indicate significant differences (*p* < 0.05) for belowground components. Vertical bars show S.D. *n* = 9.

In aboveground biomass, the mean concentrations of C, N, and P ranged from 403.56 to 424.98 g kg^−1^, 11.98 to 19.37 g kg^−1^, and 1.02 to 1.24 g kg^−1^, respectively (Figure 1). The C concentration was significantly higher under EG than under GG and MG, whereas GG had the highest N concentration (*p* < 0.05) (Figure 1A,B). In contrast, EG and MG had significantly lower aboveground P concentrations than GG (*p* < 0.05) (Figure 1C). For belowground biomass, average C, N, and P concentrations were 404.47–426.22, 9.65–14.78, and 0.76–0.92 g kg^−1^, respectively. The trends in belowground C and N concentrations across management strategies were consistent with those observed aboveground (Figure 1A,B). Notably, MG had a significantly lower P concentration than both EG and GG (*p* < 0.05) (Figure 1C).

Under the three management strategies, there were significant variations in aboveground C:N ratios (21.5–31.8) and belowground C:N ratios (28.3–42.1) among the three management strategies (*p* < 0.05) (Figure 1D). The aboveground C:P ratio peaked in EG, whereas the belowground C:P ratio was highest in MG (*p* < 0.05) (Figure 1E). The aboveground N:P ratios ranged from 11.8 to 15.6, while the belowground ratios ranged from 13.0 to 16.7 (Figure 1F). For aboveground, the maximum N:P ratio was observed in GG, whereas belowground N:P ratios did not differ significantly across the three management strategies.

### 2.3. Correlations of C, N, and P Concentrations in Plant–Soil System

**Figure 2:**
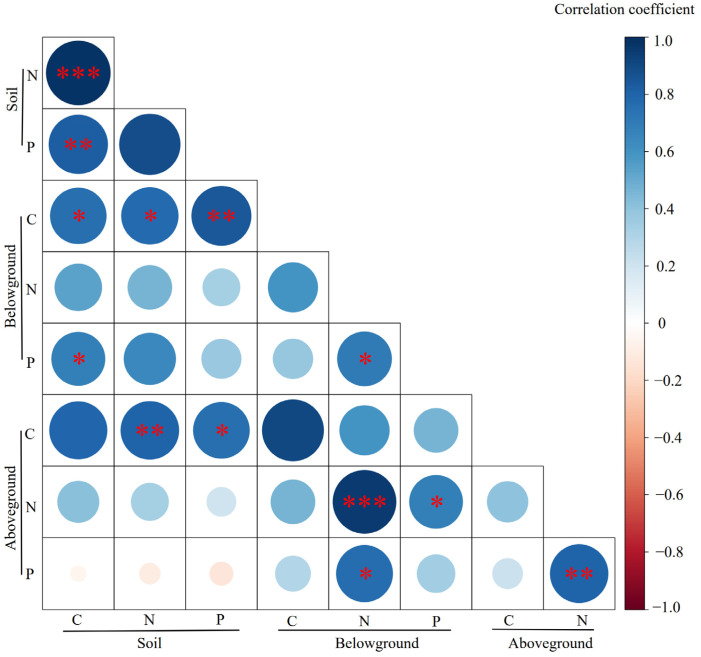
Pearson’s correlation coefficients between plant and soil C, N, and P concentrations. ***, **, and * represent *p* < 0.001, *p* < 0.01, and *p* < 0.05, respectively.

The correlation coefficients for plant and soil C, N, and P concentrations are shown in Figure 2. Among the soil elements, soil C was significantly and positively correlated with both N and P concentrations (*p* < 0.01), while no significant correlation was observed between soil N and P. In terms of nutrients in plant aboveground and belowground biomass, aboveground N was significantly and positively correlated with belowground N (*p* < 0.001) and with belowground P (*p* < 0.05); and aboveground P demonstrated a significant positive correlation with belowground N (*p* < 0.05). Within the soil–plant system, soil C was significantly and positively correlated with C and P in belowground biomass (*p* < 0.05); soil N content exhibited significant positive correlations with C in both belowground and aboveground biomass (*p* < 0.05, *p* < 0.01); and soil P content was significantly and positively associated with C content in both belowground and aboveground biomass (*p* < 0.01, *p* < 0.05).

### 2.4. Stocks of C, N, and P Under Different Management Strategies

Aboveground C, N, and P stocks range from 53.58 to 77.08 g m^−2^, 1.89 to 2.94 g m^−2^, and 0.16 to 0.19 g m^−2^, respectively (Table 2). Enclosing significantly increased the C, N, and P stocks in aboveground biomass (*p* < 0.05). Belowground C, N, and P stocks were 44.91–92.83, 1.60–2.93, and 0.10–0.20 g m^−2^, respectively, with trends across management strategies consistent with those observed aboveground. Among all strategies, soil C, N, and P stocks were highest under EG due to the enclosing treatment. At ecosystem level, total C and N stocks were significantly higher in EG than in both GG and MG (*p* < 0.05), while P stocks did not differ significantly across the three strategies. C, N, and P were predominantly sequestered in the soil, accounting for 97.6–99.9% of the total in the grassland.

## 3. Discussion

### 3.1. Effects of Management Strategies on Soil C, N, P

Soil serves as the primary substrate for the growth of terrestrial plants [26]. Biological processes, such as decaying biomass and animal excrement, constitute the main sources of soil C, N, and P [27,28]. Moreover, P is also supplied by mechanical weathering of rocks and remains relatively stable in terms of P stocks across different soil layers [29]. Under all three management strategies, we observed a progressive decline in soil C, N, and P concentrations with increasing soil depth (Table 1). This pattern aligns with previous findings [30,31] and is due to the accumulation of organic matter and root exudates in topsoil layers [32].

Our results revealed that EG was an effective practice to enhance soil C, N, and P concentrations and stocks (Table 1 and Table 2), indicating that enclosing is an effective way to accumulate more soil nutrients in the study region. These results are in accordance with other grassland studies [19,33,34] and could be attributed to the following three mechanisms. First, enclosing enhances the increments of aboveground biomass, litter mass, and root mass into the soil [20]. Second, improved surface soil moisture due to greater plant height and canopy cover boosts net primary productivity and then accelerates organic matter input to soil [23]. Third, enhanced plant coverage reduces soil exposure to wind erosion, mitigating C loss [35]. Conversely, contrasting evidence suggests that grazing can elevate soil C and N stocks by promoting root turnover, litter decomposition via trampling, and nutrient return through urine and dung, thereby facilitating nutrient cycling and organic matter incorporation [22,26]. Overall, the effects of enclosing on soil nutrients are time-dependent, often diminishing over the long term, while mowing impacts tend to be surface-limited and potentially negative at depth. For instance, Chen et al. [36] reported that mowing significantly reduced soil C concentration at 50–70 cm depth, likely due to reduced root inputs and altered C allocation patterns under repeated biomass removal without excreta returns. Across management types, P concentrations and stocks are the most stable, largely unaffected by grazing, mowing, or enclosure [26,37], reflecting the geogenic origin and low mobility of phosphorus [38].

In soils, the stoichiometric ratios of C, N, and P play a critical role in modulating nutrient availability for both plants and soil microorganisms [14,39], and these ratios often exhibit considerable variability [40,41]. In the present study, the average soil C:N, C:P, and N:P ratios were considerably lower than the global averages reported by Cleveland and Liptzin [42] and the national averages for Chinese soils [43]. Previous studies have suggested that grazing can reduce soil C:N ratios by accelerating nutrient cycling and increasing excreta inputs [4,44]. In contrast, our results indicated that the soil C:N ratio remained relatively stable across different management strategies (Table 1). This stability may be attributed to the close coupling between soil C and N cycles under different management strategies [29].

Low soil C:P ratio facilitates microbial decomposition of organic matter, leading to nutrient release and an increase in soil available P level. In contrast, a high C:P ratio can inhibit microbial decomposition, resulting in P limitation and consequently constraining plant growth [45]. In the present study, the soil C:P ratios in the 0–10 cm layer were 36.44, 37.99, 33.95 under EG, GG, and MG, respectively (Table 1). Although some variation in C:P ratios was observed among treatments, the differences were relatively small. Theoretically, a higher soil C:P ratio may suggest lower phosphorus availability or a greater potential for P limitation on microbial decomposition of organic matter [29]. However, since direct measurements of soil available P, microbial activity, or decomposition rates are not available in this study, such interpretations remain speculative. Therefore, we cautiously conclude that the observed changes in soil C:P ratios only provide preliminary indications of potential differences in soil P cycling under different management strategies, and further investigations are needed to clarify the underlying mechanisms.

The average N:P ratios ranged from 2.50 to 3.16 across treatments, which were lower than those reported for the Loess Plateau (3.62) [46] and for Chinese soils overall (3.9) [43]. Our results are consistent with this threshold. Notably, EG displayed significantly higher N:P ratios throughout the entire soil depth compared with GG and MG (*p* < 0.05) (Table 1), a pattern primarily attributed to the elevated N concentrations resulting from enclosure management.

### 3.2. Effects of Management Strategies on C, N, P

In this study, EG raised plant C concentration while lowering N concentration (Figure 1A,B). This result aligns with earlier research findings [22,47], which indicated that plants under EG accumulated more structural C compounds but faced reduced N availability due to the absence of animal excreta and slower mineralization compared to GG [2,22]. GG substantially raised the concentrations of N and P in plant tissues, according to our results (Figure 1B,C), which aligned well with previous studies in other grasslands [2,48]. For instance, Heyburn et al. [2] demonstrated that grazing significantly increased N and P concentrations in both aboveground and belowground plant compartments, primarily due to accelerated nutrient cycling via dung and urine deposition. Similarly, Schönbach et al. [4] also showed that grazing stimulated plant regrowth and nutrient uptake. The increase in N and P concentrations might be attributed to the following two reasons. First, grazing animals can boost plant growth and commonly elevate shoot N levels [30]. Second, the accelerated N turnover resulting from trampling and excreta input can raise available N in free-grazing plots [49]. MG effects on plant nutrient concentrations significantly differed from EG and GG (Figure 1), which could be due to the absence of excreta returns [50,51].

The observed differences in plant C:N:P stoichiometry across the three grassland management modes can be influenced by species composition, nutrient availability, and physiological status, which are driven by long-term management practices [52]. In this study, although MG showed the highest C:N ratio (Figure 1D), it also had the lowest biomass C and N concentrations. This suggests that the elevated C:N ratio under MG likely results from a greater reduction in N than in C, possibly due to repeated biomass removal or species shifts, rather than enhanced C assimilation. GG reduced C:N and C:P ratios compared with EG (Figure 1D,E), which may indicate relatively higher N and P availability under grazing, but this interpretation remains speculative without direct measurements of soil available nutrients or plant nutrient uptake. Consistent with previous studies [36,53], management did not significantly alter belowground N:P ratios (Figure 1F), possibly due to root stoichiometric homeostasis [54]. Further investigations are needed to clarify the physiological mechanisms underlying these stoichiometric shifts.

Plant tissue N:P ratio serves as a reflection of the relative availability of N versus P to plants, and is considered a straightforward metric for assessing nutrient limitation status across broad spatial scales [55]. In general, N:P ratios exceeding 16 suggest P limitation, those falling between 14 and 16 imply co-limitation by N and P, and values below 14 point to N limitation [56]. In the present study, the N:P ratios of aboveground biomass under EG and GG were 15.3 and 15.6, respectively, indicating that N and P jointly limited plant growth under these two treatments. By contrast, the N:P ratio in MG was 11.8, suggesting that N limitation was stronger than P limitation for plant growth under mowing. However, findings from alpine grasslands on the Qinghai-Tibetan Plateau indicated that P availability limits plant growth more strongly than does N [57,58]. These differences in N:P ratios can be attributed to differences in nutrient cycles and corresponding soil nutrients [59,60].

Our study found that plant C and P stocks followed the order EG > MG > GG, while N stocks followed EG > GG ≈ MG (Table 1). Due to the limited livestock grazing in EG, a substantial amount of fresh biomass—including green plants, root residues, and root exudate—was returned to the soil each year. This greater organic matter input ultimately resulted in higher plant C, N, P stocks in EG compared to MG and GG. The observed reduction in plant C, N, and P stocks under grazing aligns with previous findings [10,44]. Grazing by livestock can reduce nutrient retention in plant pools through two primary mechanisms. First, grazing removes substantial plant biomass via direct consumption, thereby diminishing the standing nutrient pools in aboveground vegetation [61]. Second, trampling and defoliation associated with grazing can impair root development or induce greater allocation of C to shoots for leaf regrowth, potentially limiting nutrient uptake from soil [10,62]. Conversely, Zheng et al. [48] reported that grazing enhanced nutrient stocks in a meadow steppe, attributed to increases in both plant biomass and tissue nutrient concentrations. These contradictory findings likely stem from differences in vegetation type or site-specific conditions [63], highlighting the need for further research to clarify nutrient dynamics across diverse regions and under varying management strategies.

### 3.3. Relationships Between Plant and Soil C, N, and P

In the soil, C exhibited strong positive correlations with both N and P (Figure 2). Consistent with earlier observations from Tibetan Plateau grasslands [52], soil C was tightly linked to N, suggesting that the decomposition of plant litter serves as a key process for the recovery of soil C and N [64,65]. However, no significant correlation was found between soil N and P, implying that the accumulation and cycling of these two elements might be decoupled in the studied grasslands, possibly due to distinct retention mechanisms under different management strategies.

In plant biomass, aboveground N concentration was significantly positively correlated with belowground N and P, and aboveground P was positively correlated with belowground N, which was consistent with findings from previous research [57]. These cross-correlations indicate a coordinated nutrient allocation strategy between aboveground and belowground tissues [60]. Plants appear to maintain synchronous N status across organs, and also link belowground N with aboveground P accumulation, possibly reflecting the close functional integration of roots and shoots in nutrient uptake and photosynthetic metabolism [66].

Although many studies have demonstrated strong relationships among C, N and P in soils or in plants [5,57], few have shown how the concentrations of these elements in soil relate to their concentrations in plants under different management strategies. Our results demonstrated multiple significant relationships between soil and plant nutrients (Figure 2). Soil C showed a positive relationship with belowground C and P, highlighting the role of soil organic matter in supplying carbon substrates and energy for root growth and nutrient uptake, as well as potentially influencing root P acquisition [67]. Soil N was positively associated with plant C in both belowground and aboveground biomass. This could be interpreted as an effect of soil N availability on plant productivity, that higher soil N generally promotes plant growth and, thus, increases biomass C accumulation [37]. Similarly, soil P was positively associated with plant C in both biomass compartments. This positive association between soil P and plant C likely reflects that greater P supply enhances photosynthesis and biomass production [64].

### 3.4. Limitations of the Present Study

The present study provided important insights into the effects of management strategies on C:N:P stoichiometry and stocks. However, several limitations should also be considered. First, the research was carried out in a specific region of semi-arid grasslands in North China, which may constrain the generalizability of the findings to other grassland ecosystems under different geographic and climatic conditions. Second, how species composition influenced plant and soil C, N, and P stoichiometries was not investigated in this study, even though different grassland management strategies applied at our study sites could have induced shifts in plant species composition, thereby affecting the observed stoichiometric patterns. Third, this study did not evaluate the contribution of microbial communities to mediating nutrient cycling. Microorganisms in the soil are key drivers of C, N, and P transformations, and understanding their response to different management strategies could provide deeper insights into the mechanisms governing plant and soil interactions. Fourth, the soil granulometric composition was not measured in this study, which may constrain a full understanding of soil C, N, and P storage capacity. Nevertheless, our measured soil properties (e.g., organic C, total N, total P, and stoichiometric ratios) still provide valuable information on soil nutrients research under different grassland management strategies. Future research should prioritize identifying the key drivers of nutrient cycling and determining the optimal duration of enclosure for maximizing nutrient sequestration in grasslands.

## 4. Materials and Methods

### 4.1. Overview of the Study Area

**Figure 3:**
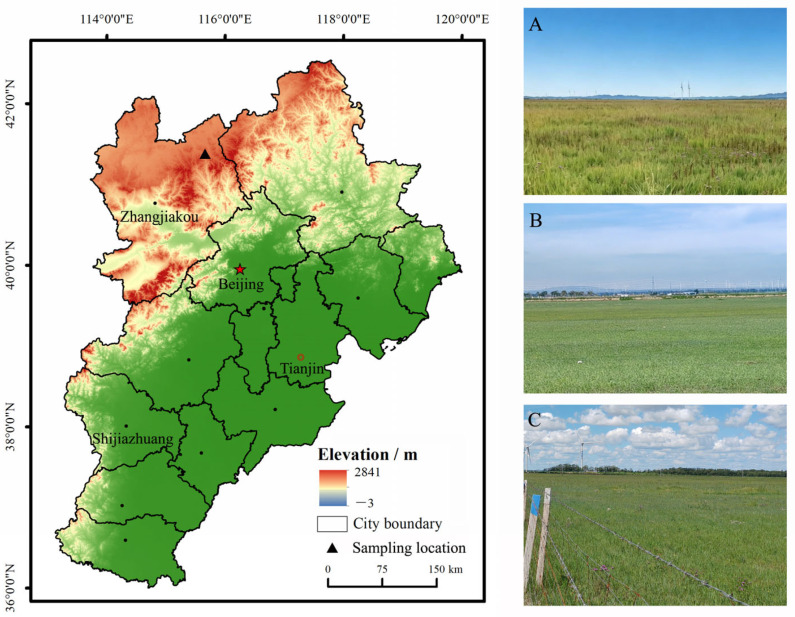
Geographical location of the study site and sampling sites of different management strategies in Hebei Province, North China. (**A**) enclosure grassland (EG); (**B**) GG, grazing grassland (gg); (**C**) Mowing grassland (MG).

The present study was conducted in Guyuan County (41°14′–41°56′ N, 114°50′–116°04′ E), located in the traditional APTZNC. The county lies to the north of Zhangjiakou city in northern Hebei Province (Figure 3). The region is characterized by a temperate continental climate, with elevations ranging from 1356 to 2123 m above sea level. The average annual temperature is 2.1 °C, with maximum and minimum temperatures of 36.1 °C in July and −39.9 °C in January, respectively. Mean annual precipitation is about 425 mm, with the majority falling during the summer months. The landscape of this region consists of flat, expansive terrain interspersed with rolling hills [63]. The soils belong to the chestnut type (Calcic Kastanozems), corresponding to Calcic-orthic Aridisol according to the U.S. soil taxonomy system [63].

### 4.2. Field Sampling and Measurement

Three grassland management strategies were implemented in 2019 under identical environmental conditions. The field survey was undertaken in late August 2025. For each management type, three 20 × 20 m sample plots were randomly established. The basic characteristics of the sample plots are shown in Table 3. Within each plot, three 25 × 25 cm PVC frame quadrats were placed along the diagonal (at the midpoint and both ends). Within each quadrat, all aboveground plant material (including both green plants and litter) was cut flush to the ground, collected, oven-dried at 65 °C for 48 h, and subsequently weighed. To estimate total belowground biomass, three root cores (5 cm in diameter) were collected from each quadrat using a soil auger. The three cores from each quadrat were combined and washed with water on a 2 mm mesh sieve to discard soil and organic particles. The purified root samples were then dried at 65 °C for 48 h to constant mass before weighing [2,7].

Soil samples were obtained using a soil auger (5 cm in diameter) from four soil layers (0–10, 10–20, 20–30, and 30–40 cm) at each sampling point, with three replicates per layer. A total of 324 soil samples (3 management types × 3 plots × 3 quadrates × 4 layers × 3 replicates) were collected. In the laboratory, the gathered soil samples underwent air-drying, grinding, and sieving prior to subsequent experimentation [32]. Additionally, three soil cores (100 cm^3^ volume) were randomly collected from each soil layer to determine the soil bulk density (BD) (g cm^−3^) by the volumetric ring method [5].

Plant and soil samples were ground to pass through a 0.2 mm sieve before analysis of their C, N, and P contents. Organic C was determined using the H_2_SO_4_-K_2_Cr_2_O_7_ oxidation method [22]. After digestion with H_2_SO_4_–H_2_O_2_, total nitrogen (TN) and total phosphorus (TP) concentration were analyzed by the micro-Kjeldahl and colorimetric (UV spectrophotometer) methods, respectively [68].

### 4.3. Data Calculation

In this study, the stocks (g m^−2^) of C, N, and P in the plant system (including aboveground and belowground) were calculated as follows [69]:

Biomass *C*/*N*/*P* Stock = *BOC*/*BTN*/*BTP* × *Bio* × 10^−3^

where *BOC*, *BTN*, and *BTP* represent the C, N and P concentrations (g kg^−1^) of the respective plant parts (aboveground and belowground), and *Bio* is the biomass of those plant parts (kg m^−2^).

The SOC, TN, and TP stocks (g m^−2^) of soil were computed using the following equation [44]:


$${SOC/TN/TP}_{\mathrm{Stock}}=\sum_{i=1}^{n}{{SOC}_{i}/{TN}_{i}/TP}_{i}\times {BD}_{i}\times D_{i}\times 10$$

where *SOC_i_*, *TN_i_*, and *TP_i_* are the concentrations of SOC, TN, and TP in layer *i* (g kg^−1^), respectively; *BD_i_* is the soil bulk density in layer *i* (g cm^−3^); *D_i_* is the thickness (cm) for layer *i*, and 10 is the unit conversion factor.

### 4.4. Statistical Analysis

One-way analysis of variance (ANOVA) followed by Duncan’s test was employed to examine the effects of the different management strategies on plant characteristics and soil properties. All statistical computations were performed with SPSS 18.0 (SPSS Inc., Chicago, IL, USA) software. The figures were generated using Microsoft Excel 2019 (Microsoft Inc., Redmond, WA, USA). Pearson correlation coefficients were used to determine the correlations between the plant characteristics and soil characteristics. Correlation analysis and plotting were performed using the “corrplot” package [70] in R 4.3.3 software.

## 5. Conclusions

Enclosing proved most effective for enhancing C, and N concentrations in both vegetation and soils, leading to the highest ecosystem C and N stocks. Soil C:N ratios remained stable (<12) across management strategies. Strong coupling among C, N, and P in the plant–soil system was evident, though total P stocks were unaffected by management, reflecting P’s geogenic origin. These findings are critical for assessing the effects of management strategies on ecosystem C, N, and P stoichiometry and stocks in semi-arid regions, and for comprehending the sophisticated relationships between plant and soil nutrients.

## Figures and Tables

**Table 1:** Soil carbon (C), nitrogen (N), phosphorus (P) concentrations and stoichiometric characteristics of different management strategies.

Management Modes	Soil Depth (cm)	C (g kg^−1^)	N (g kg^−1^)	P (g kg^−1^)	C:N	C:P	N:P
EG	0–10	20.68 ± 0.61 a	2.16 ± 0.08 a	0.57 ± 0.03 a	9.57 ± 0.51 a	36.44 ± 1.59 ab	3.81 ± 0.20 a
	10–20	16.77 ± 0.97 a	1.94 ± 0.10 a	0.51 ± 0.01 a	8.66 ± 0.18 a	32.87 ± 2.03 a	3.80 ± 0.24 a
	20–30	9.32 ± 1.32 a	0.93 ± 0.05 a	0.38 ± 0.02 a	10.02 ± 1.44 a	26.30 ± 4.41 a	2.62 ± 0.09 a
	30–40	6.54 ± 0.88 a	0.73 ± 0.02 a	0.30 ± 0.01 a	8.97 ± 1.38 a	21.57 ± 3.03 a	2.41 ± 0.06 a
GG	0–10	18.26 ± 0.76 b	1.79 ± 0.09 b	0.48 ± 0.03 b	10.2 ± 1.21 a	37.99 ± 1.24 a	3.72 ± 0.10 b
	10–20	12.09 ± 0.94 b	1.36 ± 0.08 b	0.48 ± 0.04 ab	8.86 ± 0.98 a	25.34 ± 1.42 b	2.86 ± 0.08 b
	20–30	8.02 ± 0.71 ab	0.75 ± 0.05 b	0.36 ± 0.02 a	10.77 ± 1.36 a	22.2 ± 1.48 a	2.07 ± 0.15 b
	30–40	5.79 ± 0.28 ab	0.63 ± 0.03 b	0.28 ± 0.02 a	9.15 ± 0.79 a	20.54 ± 1.23 ab	2.25 ± 0.16 b
MG	0–10	16.54 ± 0.96 c	1.70 ± 0.06 b	0.49 ± 0.01 b	9.73 ± 0.70 a	33.95 ± 1.78 b	3.49 ± 0.07 b
	10–20	10.83 ± 0.61 b	1.21 ± 0.13 b	0.44 ± 0.01 b	9.00 ± 0.46 a	24.83 ± 1.68 b	2.77 ± 0.22 b
	20–30	7.35 ± 0.59 b	0.65 ± 0.09 b	0.37 ± 0.03 a	11.4 ± 1.37 a	20.25 ± 2.75 a	1.78 ± 0.22 b
	30–40	4.87 ± 0.37 b	0.55 ± 0.04 c	0.28 ± 0.02 a	8.81 ± 0.86 a	17.32 ± 1.46 b	1.97 ± 0.14 b

EG, enclosure grassland; GG, grazing grassland; MG, Mowing grassland. Values in the tables are means ± S.D. (Standard deviation). Different lowercase letters indicate significant differences among management strategies.

**Table 2:** C, N, and P stocks in various ecosystem components under management strategies.

Parameters		C Stocks (g m^−2^)			N Stocks g m^−2^)			P Stocks (g m^−2^)		
		EG	GG	MG	EG	GG	MG	EG	GG	MG
Aboveground		77.08 ± 6.20 a	53.58 ± 3.13 c	63.60 ± 4.34 b	2.94 ± 0.27 a	2.51 ± 0.24 b	1.89 ± 0.14 c	0.19 ± 0.01 a	0.16 ± 0.02 b	0.16 ± 0.01 b
Belowground		92.83 ± 8.51 a	44.91 ± 4.76 b	68.35 ± 7.19 ab	2.93 ± 0.86 a	1.60 ± 0.20 b	1.63 ± 0.21 b	0.20 ± 0.06 a	0.10 ± 0.02 b	0.13 ± 0.01 ab
Soil	0–10	2226.94 ± 41.16 a	2269.33 ± 90.56 a	1945.02 ± 87.55 b	232.68 ± 8.28 a	222.94 ± 14.02 a	200.37 ± 9.22 b	61.37 ± 2.35 a	60.07 ± 4.26 a	57.24 ± 1.79 a
	10–20	1952.83 ± 138.05 a	1523.4 ± 114.15 b	1339.59 ± 29.62 b	225.43 ± 14.78 a	172.21 ± 10.12 b	149.05 ± 10.72 b	59.33 ± 1.20 a	60.06 ± 5.04 a	54.04 ± 0.94 a
	20–30	1186.68 ± 159.65 a	1099.75 ± 140.29 a	932.94 ± 46.87 a	118.61 ± 8.38 a	102.23 ± 9.60 a	82.47 ± 10.03 b	45.49 ± 2.71 a	49.78 ± 5.40 a	46.64 ± 3.83a
	30–40	876.46 ± 101.93 a	827.94 ± 42.26 a	654.7 ± 28.96 b	98.08 ± 6.32 a	90.61 ± 4.81 a	74.51 ± 4.67 b	40.75 ± 2.56 a	40.54 ± 2.30 a	38.13 ± 0.62 a
Total		6412.82 ± 206.60 a	5818.91 ± 239.8 b	5004.19 ± 139.50 c	680.68 ± 9.79 a	592.11 ± 32.47 b	509.92 ± 27.64 c	207.35 ± 6.87 a	210.71 ± 11.70 a	196.33 ± 5.15 a

EG, enclosure grassland; GG, grazing grassland; MG, Mowing grassland. Values in the tables are means ± S.D. (Standard deviation). Different lowercase letters indicate significant differences among management strategies (*p* < 0.05).

**Table 3:** Plot characteristics of different management strategies in Guyuan County, Hebei Province, China.

Parameters		Management Modes		
		EG	GG	MG
Dominant species		*Leymus chinensis* (Trin.) Tzvel., *Cleistogenes squarrosa* (Trin.) Keng, *Chenopodium glaucum* Linn., etc.	*L*. *chinensis* (Trin.) Tzvel., *Potentilla lancinata* Card., *Artemisia dalai*-*lamae* Krasch., etc.	*L*. *chinensis* (Trin.) Tzvel., *Stipa grandis* P. Smirn., etc.
Coverage (%)		83.4	76.5	80.6
Height (cm)		18.7	16.2	17.2
Land overview		EG was established in 2019; before then, EG had been in a state of long- term grazing	GG has a free-grazing history > 60 years.	MG was mowed every September since 2019, with all biomass subsequently removed.
Aboveground biomass (g m^−2^)		181.4	129.6	157.6
Belowground biomass (g m^−2^)		218.5	100.9	168.9
Soil bulk density (g cm^−3^)	0–10 cm	1.08	1.24	1.18
	10–20 cm	1.16	1.26	1.24
	20–30 cm	1.27	1.37	1.27
	30–40 cm	1.34	1.43	1.35

EG, enclosure grassland; GG, grazing grassland; MG, Mowing grassland.

## Data Availability

The original contributions presented in this study are included in the article. Further inquiries can be directed to the corresponding author.

## References

[1] Bai Y., Cotrufo M.F. (2022). Grassland soil carbon sequestration: Current understanding, challenges, and solutions. Science.

[2] Heyburn J., McKenzie P., Crawley M.J., Fornara D.A. (2017). Effects of grassland management on plant C:N:P stoichiometry: Implications for soil element cycling and storage. Ecosphere.

[3] He M., Zhou G., Yuan T., Van Groenigen K.J., Shao J., Zhou X. (2020). Grazing intensity significantly changes the C:N:P stoichiometry in grassland ecosystems. Glob. Ecol. Biogeogr..

[4] Schönbach P., Wan H., Gierus M., Bai Y., Müller K., Lin L., Susenbeth A., Taube F. (2011). Grassland responses to grazing: Effects of grazing intensity and management system in an Inner Mongolian steppe ecosystem. Plant Soil.

[5] Chen L., Baoyin T., Xia F. (2022). Grassland management strategies influence soil C, N, and P sequestration through shifting plant community composition in a semi-arid grasslands of Northern China. Ecol. Indic..

[6] Abdalla M., Hastings A., Chadwick D.R., Jones D.L., Evans C.D., Jones M.B., Rees R.M., Smith P. (2018). Critical review of the impacts of grazing intensity on soil organic carbon storage and other soil quality indicators in extensively managed grasslands. Agricul. Ecosyst. Environ..

[7] Chai Q., Ma Z., Chang X., Wu G., Zheng J., Li Z., Wang G. (2019). Optimizing management to conserve plant diversity and soil carbon stock of semi-arid grasslands on the Loess Plateau. CATENA.

[8] Jiang J., Wang Y.-P., Yang Y., Yu M., Wang C., Yan J. (2019). Interactive effects of nitrogen and phosphorus additions on plant growth vary with ecosystem type. Plant Soil.

[9] Lu J., Feng S., Wang S., Zhang B., Ning Z., Wang R., Chen X., Yu L., Zhao H., Lan D. (2023). Patterns and driving mechanism of soil organic carbon, nitrogen, and phosphorus stoichiometry across northern China’s desert-grassland transition zone. CATENA.

[10] Guo Y., Boughton E.H., Emmi A.L., Anderson E., Landau L., Qiu J. (2025). Agricultural land management and grazing intensification effects on ecosystem carbon and nutrient stocks. Ecosystems.

[11] Verdoodt A., Mureithi S.M., Van Ranst E. (2010). Impacts of management and enclosure age on recovery of the herbaceous rangeland vegetation in semi-arid Kenya. J. Arid Environ..

[12] Wilson C.H., Strickland M.S., Hutchings J.A., Bianchi T.S., Flory S.L. (2018). Grazing enhances belowground carbon allocation, microbial biomass, and soil carbon in a subtropical grassland. Glob. Change Biol..

[13] Jiang Z., Hu Z., Lai D.Y.F., Han D., Wang M., Liu M., Zhang M., Guo M. (2020). Light grazing facilitates carbon accumulation in subsoil in Chinese grasslands: A meta-analysis. Glob. Change Biol..

[14] Liu C., Li W., Xu J., Wei W., Xue P., Yan H. (2021). Response of soil nutrients and stoichiometry to grazing management in alpine grassland on the Qinghai-Tibet Plateau. Soil Till. Res..

[15] Hu Y.-Y., Wei H.-W., Zhang Z.-W., Hou S.-L., Yang J.-J., Wang J.-F., Lü X.-T. (2020). Changes of plant community composition instead of soil nutrient status drive the legacy effects of historical nitrogen deposition on plant community N:P stoichiometry. Plant Soil.

[16] Hou S.-L., Yin J.-X., Sistla S., Yang J.J., Sun Y., Li Y.Y., Lü X.T., Han X.G. (2017). Long-term mowing did not alter the impacts of nitrogen deposition on litter quality in a temperate steppe. Ecol. Eng..

[17] Li W., Huang G., Zhang H. (2020). Enclosure increases nutrient resorption from senescing leaves in a subalpine pasture. Plant Soil.

[18] Abrigo M., Lezama F., Grela I., Piñeiro G. (2024). Grazing exclusion effects on vegetation structure and soil organic matter in Savannas of Río de La Plata grasslands. J. Veg. Sci..

[19] Dai L., Fu R., Guo X., Du Y., Lin L., Zhang F., Li Y., Cao G. (2021). Long-term grazing exclusion greatly improve carbon and nitrogen store in an alpine meadow on the Northern Qinghai-Tibet Plateau. CATENA.

[20] Xiong D., Shi P., Zhang X., Zou C.B. (2016). Effects of grazing exclusion on carbon sequestration and plant diversity in grasslands of China—A meta-analysis. Ecol. Eng..

[21] Shang Z., Cao J., Guo R., Henkin Z., Ding L., Long R., Deng B. (2017). Effect of enclosure on soil carbon, nitrogen and phosphorus of alpine desert rangeland. Land. Degrad. Dev..

[22] Zhang Q., Zhou D., Hu J. (2022). Effects of long-term enclosing on distributions of carbon and nitrogen in semia-arid grassland of Inner Mongolia. Ecol. Inform..

[23] Deng L., Shangguan Z.-P., Wu G.-L., Chang X.-F. (2017). Effects of grazing exclusion on carbon sequestration in China’s grassland. Earth-Sci. Rev..

[24] Yang X., Chen H., Gong Y., Zheng X., Fan M., Kuzyakov Y. (2015). Nitrous oxide emissions from an Agro-Pastoral Ecotone of Northern China depending on land uses. Agricul. Ecosyst. Environ..

[25] Huan L., Yuyan Y., Zemin A., Xiaohu D., Yong C., Qingqing L., Mengjia H., Haoli H., Yuanyuan Z., Tian C. (2025). Spatial and temporal evolution of forage-livestock balance in the Agro-Pastoral Transition Zone of Northern China. J. Arid Land.

[26] Pan Y., Tang H., Fang F., Ma Y., Chen Z. (2023). Is Elemental stoichiometry (C, N, P) of soil and soil microbial biomass influenced by management modes and soil depth in Agro-Pastoral Transitional Zone of Northern China?. J. Soil. Sediment..

[27] Peichl M., Leava N.A., Kiely G. (2012). Above- and belowground ecosystem biomass, carbon and nitrogen allocation in recently afforested grassland and adjacent intensively managed grassland. Plant Soil.

[28] Tao Y., Zhou X.-B., Zhang S.-H., Lu H.-Y., Shao H. (2020). Soil nutrient stoichiometry on linear sand dunes from a temperate desert in Central Asia. CATENA.

[29] Lu Q., Fan H., Yan B., Zhao D., Wei X. (2023). Soil C, N, and P and C:N:P stoichiometry associated with environmental factors in two typical alpine grasslands in Northern Tibet. J. Soil. Sediment..

[30] Liu N., Kan H.M., Yang G.W., Zhang Y.J. (2015). Changes in plant, soil, and microbes in a typical steppe from simulated grazing: Explaining potential change in soil C. Ecol. Monogr..

[31] Ma J., Li L.H., Guo L.P., Bai L., Zhang J.R., Chen Z.H., Ahmad S. (2015). Variation in soil nutrients in grasslands along the kunes river in Xinjiang, China. Chem. Ecol..

[32] Liu M., Shi J., Zhang X. (2025). Responses of ecological stoichiometry of plants and soils to degradation levels in alpine wetlands of the Qinghai-Tibet Plateau. Environ. Manag..

[33] Yu L., Chen Y., Sun W., Huang Y. (2019). Effects of grazing exclusion on soil carbon dynamics in alpine grasslands of the Tibetan Plateau. Geoderma.

[34] Witt G.B. (2011). Carbon sequestration and biodiversity restoration potential of semi-arid mulga lands of australia interpreted from long-term grazing exclosures. Agricul. Ecosyst. Environ..

[35] Mekuria W., Veldkamp E., Haile M., Nyssen J., Muys B., Gebrehiwot K. (2007). Effectiveness of exclosures to restore degraded soils as a result of overgrazing in Tigray, Ethiopia. J. Arid Environ..

[36] Chen L., Wang K., Baoyin T. (2021). Effects of grazing and mowing on vertical distribution of soil nutrients and their stoichiometry (C:N:P) in a semi-arid grassland of North China. CATENA.

[37] Ning Z., Zhao X., Li Y., Wang L., Lian J., Yang H., Li Y. (2021). Plant community C:N:P stoichiometry is mediated by soil nutrients and plant functional groups during grassland desertification. Ecol. Eng..

[38] Liu X., Ma J., Ma Z.W., Li L.H. (2017). Soil nutrient contents and stoichiometry as affected by land-use in an Agro-Pastoral Region of Northwest China. CATENA.

[39] Vitousek P.M., Farrington H. (1997). Nutrient limitation and soil development: Experimental test of a biogeochemical theory. Biogeochemistry.

[40] Bing H., Wu Y., Zhou J., Sun H., Luo J., Wang J., Yu D. (2016). Stoichiometric variation of carbon, nitrogen, and phosphorus in soils and its implication for nutrient limitation in alpine ecosystem of Eastern Tibetan Plateau. J. Soil. Sediment..

[41] Bui E.N., Henderson B.L. (2013). C:N:P stoichiometry in australian soils with respect to vegetation and environmental factors. Plant Soil.

[42] Cleveland C.C., Liptzin D. (2007). C:N:P stoichiometry in soil: Is there a “Redfield Ratio” for the microbial biomass?. Biogeochemistry.

[43] Tian H., Chen G., Zhang C., Melillo J.M., Hall C.A.S. (2010). Pattern and variation of C:N:P ratios in China’s soils: A synthesis of observational data. Biogeochemistry.

[44] Lu X., Yan Y., Sun J., Zhang X., Chen Y., Wang X., Cheng G. (2015). Carbon, nitrogen, and phosphorus storage in alpine grassland ecosystems of Tibet: Effects of grazing exclusion. Ecol. Evol..

[45] Spohn M. (2020). Increasing the organic carbon stocks in mineral soils sequesters large amounts of phosphorus. Glob. Change Biol..

[46] Ma R., Hu F., Liu J., Wang C., Wang Z., Liu G., Zhao S. (2020). Shifts in soil nutrient concentrations and C:N:P stoichiometry during long-term natural vegetation restoration. Peer J..

[47] Liu Y., Geng X., Wei D., Dai D., Xu R. (2020). Grazing exclusion enhanced net ecosystem carbon uptake but decreased plant nutrient content in an alpine steppe. CATENA.

[48] Zheng S., Ren H., Li W., Lan Z. (2012). Scale-dependent effects of grazing on plant C:N:P stoichiometry and linkages to ecosystem functioning in the Inner Mongolia Grassland. PLoS ONE.

[49] Zhang C., Liu G., Song Z., Wang J., Guo L. (2018). Interactions of soil bacteria and fungi with plants during long-term grazing exclusion in semiarid grasslands. Soil Biol. Biochem..

[50] Tälle M., Deák B., Poschlod P., Valkó O., Westerberg L., Milberg P. (2016). Grazing vs. mowing: A meta-analysis of biodiversity benefits for grassland management. Agricul. Ecosyst. Environ..

[51] Zhu Y., Delgado-Baquerizo M., Shan D., Yang X., Eldridge D.J. (2021). Grazing impacts on ecosystem functions exceed those from mowing. Plant Soil.

[52] Du C., Gao Y. (2021). Grazing exclusion alters ecological stoichiometry of plant and soil in degraded alpine grassland. Agric. Ecosyst. Environ..

[53] Liu J., Li L., Ji L., Li Y., Liu J., Li F.Y. (2023). Divergent effects of grazing versus mowing on plant nutrients in typical steppe grasslands of Inner Mongolia. J. Plant Ecol..

[54] Kobiela B., Biondini M., Sedivec K. (2016). Comparing root and shoot responses to nutrient additions and mowing in a restored semi-arid grassland. Plant Ecol..

[55] Bracken M.E.S., Hillebrand H., Borer E.T., Seabloom E.W., Cebrian J., Cleland E.E., Elser J.J., Gruner D.S., Harpole W.S., Ngai J.T. (2015). Signatures of nutrient limitation and co-limitation: Responses of autotroph internal nutrient concentrations to nitrogen and phosphorus additions. Oikos.

[56] Reich P.B., Oleksyn J. (2004). Global patterns of plant leaf n and p in relation to temperature and latitude. Proc. Natl. Acad. Sci. USA.

[57] Yan Y., Lu X. (2020). Are N, P, and N:P stoichiometry limiting grazing exclusion effects on vegetation biomass and biodiversity in alpine grassland?. Glob. Ecol. Conserv..

[58] Song Z., Liu H., Zhao F., Xu C. (2014). Ecological Stoichiometry of N:P:Si in China’s Grasslands. Plant Soil.

[59] Hong J., Wang X., Wu J. (2014). Stoichiometry of root and leaf nitrogen and phosphorus in a dry alpine steppe on the Northern Tibetan Plateau. PLoS ONE.

[60] Güsewell S. (2004). N:P Ratios in terrestrial plants: Variation and functional significance. New Phytol..

[61] Silveira M.L., Liu K., Sollenberger L.E., Follett R.F., Vendramini J.M.B. (2013). Short-term effects of grazing intensity and nitrogen fertilization on soil organic carbon pools under perennial grass pastures in the Southeastern USA. Soil Biol. Biochem..

[62] Bai W., Fang Y., Zhou M., Xie T., Li L., Zhang W.H. (2015). Heavily intensified grazing reduces root production in an Inner Mongolia temperate steppe. Agricul. Ecosyst. Environ..

[63] Xu M., Xie F., Wang K. (2014). Response of vegetation and soil carbon and nitrogen storage to grazing intensity in semi-arid grasslands in the Agro-Pastoral Zone of Northern China. PLoS ONE.

[64] Elser J.J., Bracken M.E.S., Cleland E.E., Gruner D.S., Harpole W.S., Hillebrand H., Ngai J.T., Seabloom E.W., Shurin J.B., Smith J.E. (2007). Global analysis of nitrogen and phosphorus limitation of primary producers in freshwater, marine and terrestrial ecosystems. Ecol. Lett..

[65] Elser J.J., Fagan W.F., Kerkhoff A.J., Swenson N.G., Enquist B.J. (2010). Biological stoichiometry of plant production: Metabolism, scaling and ecological response to global change. New Phytol..

[66] Elser J.J., Fagan W.F., Denno R.F., Dobberfuhl D.R., Folarin A., Huberty A., Interlandi S., Kilham S.S., McCauley E., Schulz K.L. (2000). Nutritional constraints in terrestrial and freshwater food webs. Nature.

[67] Wu G., Gao J., Li H., Ren F., Liang D., Li X. (2023). Shifts in plant and soil C, N, and P concentrations and C:N:P stoichiometry associated with environmental factors in alpine marshy wetlands in West China. CATENA.

[68] Zhang K., Su Y., Yang R. (2019). Variation of soil organic carbon, nitrogen, and phosphorus stoichiometry and biogeographic factors across the desert ecosystem of Hexi Corridor, Northwestern China. J. Soil. Sediment..

[69] Zhang Z., Han J., Yin H., Xue J., Jia L., Zhen X., Chang J., Wang S., Yu B. (2022). Assessing the effects of different long-term ecological engineering enclosures on soil quality in an alpine desert grassland area. Ecol. Indic..

[70] Wei T.Y., Simko V. R Package ‘Corrplot’: Visualization of a Correlation Matrix (Version 0.95). https://github.com/taiyun/corrplot.

